# A Clinical and Operational Preparedness Framework for Dengue in Colombia: Integrating Surveillance, Clinical Response, Prevention, and Health System Planning

**DOI:** 10.7759/cureus.112337

**Published:** 2026-07-09

**Authors:** Alejandro Rodriguez Bobadilla, Paola Andrea Mejía Ladino, Sergio Alonso Bobadilla Ocampo, Ingrid Carolina Bobadilla Ocampo, Juan José Hennessey Hernandez

**Affiliations:** 1 Infectious Diseases, Universidad de Compensar, Bogotá, COL; 2 Biomedical Engineering, Universidad de Compensar, Bogotá, COL; 3 General Practice, Fundación Universitaria Juan N. Corpas, Bogotá, COL

**Keywords:** clinical triage, colombia, dengue, dengue vaccination, economic burden, epidemiological surveillance, health system preparedness, vector control

## Abstract

Dengue is an arboviral disease of major public health relevance in Colombia, where recurrent endemic-epidemic transmission intersects with climatic variability, urbanization, population mobility, co-circulation of viral serotypes and lineages, seasonal healthcare pressure, and substantial direct and indirect costs. Although dengue prevention and response in Colombia include epidemiological surveillance, clinical diagnosis, management of warning signs, vector-control activities, outbreak response, and assessment of preventive interventions, these components may be implemented unevenly across territories and may remain insufficiently integrated with health system planning, operational readiness, and economic risk assessment.

This technical report proposes an evidence-informed conceptual preparedness framework for dengue in Colombia. The framework was developed through a structured narrative synthesis of peer-reviewed evidence, preparedness-oriented guidance, and Colombia-specific epidemiological, clinical, operational, and health system considerations. It is intended to complement, rather than replace, existing dengue surveillance, prevention, and response strategies by organizing decision points across five interrelated domains: surveillance-to-action, risk-stratified clinical triage, adaptive vector control, contextual vaccination assessment, and healthcare response capacity. A cross-cutting economic risk-mapping component is included to identify relevant cost domains and potential pathways of preventable burden related to outpatient care, hospitalization, productivity loss, outbreak response, severe disease, and healthcare service saturation, without constituting a formal economic evaluation.

The framework emphasizes territorial prioritization, timely translation of risk signals into action, early recognition of patients at risk of progression, alignment between community-based interventions and healthcare capacity, and contextual assessment of emerging tools such as tetravalent dengue vaccines and Wolbachia-based vector strategies. It also recognizes implementation constraints, including data interoperability, laboratory and genomic capacity, workforce availability, territorial inequities, financing, pharmacovigilance, governance, and feasibility of local adoption. Future evaluation should assess operational indicators such as signal-to-alert time, triage performance, severe dengue incidence, hospitalization, healthcare resource utilization, stock availability, equity, and cost-effectiveness. In heterogeneous endemic settings, this conceptual framework may support more coordinated dengue preparedness; however, its operational usefulness, economic value, scalability, and policy relevance require stakeholder validation, territorial adaptation, pilot implementation, and prospective evaluation.

## Introduction

Dengue is a systemic arboviral infection caused by four antigenically distinct dengue virus serotypes (DENV-1, DENV-2, DENV-3, and DENV-4), transmitted primarily by Aedes mosquitoes. Its public health relevance has increased because endemic transmission, epidemic amplification, urbanization, human mobility, climatic variability, vector expansion, and co-circulation of serotypes can converge to generate recurrent pressure on surveillance systems, emergency departments, inpatient services, laboratories, and vector-control programs [1]. International preparedness guidance has emphasized that dengue prevention and control require a shift from predominantly reactive outbreak response toward integrated, anticipatory, and coordinated strategies linking surveillance, early warning, clinical management, vector control, risk communication, intersectoral action, and health system readiness [2-4]. In this context, dengue preparedness requires more than case notification or reactive vector control; it requires structured coordination between epidemiological intelligence, clinical triage, community-level interventions, healthcare capacity, governance, and health system planning.

In Colombia, dengue represents a persistent and heterogeneous public health challenge. The country has recurrent endemic-epidemic transmission, territorial differences in incidence and healthcare access, and substantial economic consequences for patients, caregivers, insurers, healthcare providers, and public health authorities [5]. The economic burden is not limited to hospitalization; it also includes outpatient care, diagnostic testing, direct non-medical expenses, productivity loss, absenteeism, premature mortality, vector-control activities, and institutional outbreak response [5]. Within a preparedness framework, these economic considerations are best interpreted as risk-mapping inputs that help identify potential cost domains for future formal evaluation, rather than as evidence of cost reduction attributable to any specific intervention.

Colombia has an established dengue surveillance and response architecture, including mandatory public health notification through the national surveillance system, event-specific surveillance protocols, epidemiological reporting, laboratory confirmation pathways, risk analysis, entomological surveillance, vector-control actions, community-oriented prevention activities, and national or territorial response guidance during periods of increased transmission [6,7]. These components constitute an essential baseline for dengue control and provide an institutional platform for preparedness. However, their operational performance may vary across territories because of differences in reporting timeliness, diagnostic capacity, laboratory access, entomological infrastructure, workforce availability, municipal field capacity, referral networks, financing, health service saturation, geographic access, and data interoperability. Consequently, the central challenge is not only the existence of dengue control components, but the degree to which they are integrated, timely, territorially prioritized, and translated into actionable decisions before and during epidemic amplification.

International frameworks provide useful operational principles but require contextual adaptation. Current World Health Organization guidance on dengue and other Aedes-borne arboviruses emphasizes preparedness, readiness, coordinated response, surveillance, clinical care, vector control, risk communication, community protection, and health system readiness [2]. The Early Warning and Response System for dengue outbreaks provides a structured approach for using surveillance data and alarm indicators to anticipate outbreaks and support response planning [3]. In the Region of the Americas, the Pan American Health Organization (PAHO) Integrated Management Strategy for Arboviral Disease Prevention and Control in the Americas has promoted a management model based on patient care, social communication, environmental management, integrated vector management, laboratory support, and epidemiological surveillance [4]. These frameworks are directly relevant to Colombia, but their local operational translation requires consideration of territorial heterogeneity, health system fragmentation, climatic and ecological variability, differential access to care, and the feasibility of implementing data-driven preparedness tools at the subnational level.

Recent Colombian evidence further supports strengthening virological and genomic surveillance as complementary inputs for public health decision-making. During the 2023-2024 increase in dengue cases in Valle del Cauca, the simultaneous circulation of multiple serotypes and lineages suggested that epidemic magnitude may reflect interactions among climatic, ecological, population-level, immunological, and viral factors rather than a single dominant lineage [8]. The detection of emerging genotypes, including the DENV-2 cosmopolitan genotype, further reinforces the relevance of linking epidemiological surveillance, molecular characterization, and territorial analysis when prioritizing higher-risk areas [9]. However, genomic surveillance is not uniformly available across Colombia, and its operational use depends on laboratory infrastructure, trained personnel, sample referral pathways, data integration, sequencing capacity, bioinformatics support, and timely translation of results into public health action.

Climate and environmental signals are also relevant to dengue preparedness. Recent studies indicate that the population exposed to climatically suitable conditions for dengue transmission has increased, and that climate variability phenomena such as the El Niño-Southern Oscillation may influence the timing, intensity, and geographic distribution of dengue risk [10,11]. These signals may inform preparedness planning, but they should not be interpreted as stand-alone predictors of local outbreaks. Their operational usefulness depends on integration with incidence trends, diagnostic positivity, case clustering, entomological information, population density, water-storage practices, urban vulnerability, vector suitability, and healthcare service pressure. Therefore, climate-informed preparedness should be understood as one input within a broader decision architecture, rather than as a deterministic forecasting instrument.

From a clinical perspective, risk-stratified triage remains one of the most actionable components of preparedness. Classification into dengue without warning signs, dengue with warning signs, and severe dengue supports clinical decision-making, but its operational value depends on timely recognition of patients at increased risk of deterioration, particularly during the critical phase of illness. Available evidence associates severe dengue with comorbidity, persistent vomiting, abdominal pain, fluid accumulation, mucosal or gastrointestinal bleeding, lethargy, hepatomegaly, hemoconcentration with thrombocytopenia, elevated transaminases, and other clinical or laboratory markers of progression [12]. Early biomarkers related to inflammation, hepatic injury, and endothelial dysfunction may provide additional prognostic information [13], but their incorporation into routine triage requires local validation, affordability, acceptable turnaround time, standardized interpretation, and clear incremental clinical utility over available clinical and laboratory variables.

Preventive interventions also require contextual interpretation. Wolbachia-infected mosquito deployments have demonstrated reductions in symptomatic dengue and dengue-related hospitalizations in a randomized community trial [14]. Tetravalent dengue vaccines, including TAK-003, have shown long-term efficacy and safety in clinical trials, although performance varies by serotype, baseline serostatus, age, and epidemiological context [15]. Modeling studies suggest that vaccination impact may be greater in moderate- to high-transmission settings and when appropriate age cohorts are selected [16,17]. These findings are relevant for preparedness planning but should not be extrapolated mechanically to Colombia without evaluating local transmission intensity, age-specific burden, seroprevalence, circulating serotypes and lineages, pharmacovigilance capacity, financing, equity, acceptability, logistics, national policy alignment, and programmatic feasibility.

This technical report presents an evidence-informed conceptual preparedness framework for dengue in Colombia. The framework is intended to complement, rather than replace, existing national and territorial dengue surveillance, prevention, and response strategies. It organizes evidence from clinical, epidemiological, virological, climatic, operational, implementation-oriented, and economic sources into five interrelated domains: surveillance-to-action, risk-stratified clinical triage, adaptive vector control, contextual vaccination assessment, and healthcare response capacity, with a cross-cutting economic risk-mapping component. Its purpose is to support structured planning, clarify operational decision points, identify feasibility constraints, and define domains for future territorial adaptation, stakeholder validation, implementation research, and prospective evaluation.

## Technical report

This technical report presents an evidence-informed conceptual preparedness framework for dengue in Colombia. The framework organizes clinical, epidemiological, virological, climatic, operational, implementation-oriented, and economic considerations into a structured planning approach for territories with recurrent dengue transmission. It is intended to complement, rather than replace, existing national and territorial dengue surveillance, prevention, and response strategies. Its purpose is to support preparedness planning, clarify operational decision points, identify feasibility constraints, and define domains that require territorial adaptation, stakeholder validation, implementation testing, and prospective evaluation.

Framework development approach

The framework was developed through a structured narrative synthesis rather than a systematic review, formal consensus process, predictive modeling exercise, or economic evaluation. The development process followed four sequential steps. First, evidence and guidance relevant to dengue preparedness were mapped across biomedical, public health, operational, and health system domains, including international dengue preparedness guidance, early warning approaches, integrated management strategies, Colombia-specific surveillance and response considerations, recent virological evidence, climate-related risk evidence, clinical triage literature, vector-control interventions, vaccination evidence, and economic burden studies [1-17]. Second, the evidence was organized into candidate preparedness functions according to their operational role: detection of risk signals, clinical risk stratification, community and vector-control response, preventive technology assessment, healthcare capacity management, and economic risk identification. Third, these functions were grouped into five interrelated domains considered necessary for a preparedness framework applicable to heterogeneous endemic settings: surveillance-to-action, risk-stratified clinical triage, adaptive vector control, contextual vaccination assessment, and healthcare response capacity. Fourth, the framework was refined conceptually by identifying feasibility constraints, suggested indicators, governance requirements, and future evaluation needs.

The framework should therefore be interpreted as a novel synthesis of existing evidence and preparedness guidance adapted to the Colombian context, rather than as a validated operational model. Its conceptual structure draws on international principles of integrated dengue prevention and control, early warning and response, and integrated management strategies, while incorporating Colombia-specific challenges related to territorial heterogeneity, surveillance implementation, laboratory and genomic capacity, health system fragmentation, differential access to care, municipal vector-control capacity, healthcare service pressure, and economic burden [2-7]. The domains were selected because they represent recurrent decision points in dengue preparedness: when to activate alerts, how to triage patients safely, where to prioritize vector-control actions, whether and how to assess preventive technologies, how to prepare healthcare services, and how to identify cost domains for future economic evaluation.

Preparedness domains

The framework is structured around five interrelated domains: surveillance-to-action, risk-stratified clinical triage, adaptive vector control, contextual vaccination assessment, and healthcare response capacity. A cross-cutting economic risk-mapping component is included to identify relevant cost domains and potential avoidable-burden pathways, but it does not constitute a formal economic evaluation. Table 1 summarizes the proposed domains, their core inputs, planning functions, evidence base, feasibility constraints, and suggested indicators.

**Table 1:** Conceptual preparedness domains for dengue in Colombia The evidence base refers to the primary type of source supporting each preparedness domain, including international preparedness guidance, national surveillance and response documents, epidemiological evidence, clinical evidence, implementation-oriented evidence, modeling studies, and expert conceptual synthesis. Suggested indicators are proposed for planning and future evaluation; they should not be interpreted as validated performance thresholds and require territorial adaptation before implementation. EWARS: Early Warning and Response System; PAHO: Pan American Health Organization

Domain	Core inputs	Planning function	Main evidence or guidance source	Feasibility constraints	Suggested indicators
Surveillance-to-action	Incidence, diagnostic positivity, hospitalizations, warning-sign visits, serotypes or lineages, climatic and environmental signals	Territorial prioritization, alert activation, laboratory readiness, supply planning, and escalation of public health response	WHO/PAHO preparedness guidance, EWARS principles, national surveillance protocols, Colombian virological evidence, climate-risk studies [2-4,6,8-11]	Data interoperability, notification timeliness, analytic capacity, governance, laboratory access, sequencing capacity, and reporting completeness	Signal-to-alert time; reporting completeness; diagnostic turnaround time; alert activation timeliness; laboratory demand
Risk-stratified clinical triage	Day of illness, warning signs, comorbidities, hydration status, vital signs, hematocrit, platelets, transaminases, and follow-up feasibility	Outpatient care, observation, admission, referral, reassessment, and patient-safety monitoring	WHO clinical classification, systematic evidence on severe dengue predictors, biomarker evidence for future validation [1,12,13]	Workforce training, laboratory access, reassessment capacity, referral barriers, standardized documentation, and safe outpatient follow-up	Warning-sign documentation; return visits; observation use; severe dengue; case fatality; referral timeliness
Adaptive vector control	Case clusters, entomological indices, water storage, environmental risk, community participation, vulnerable populations, and local vector ecology	Hotspot targeting, breeding-site reduction, larval control, selective adulticiding, community engagement, and environmental management	Integrated vector management principles, PAHO/WHO integrated strategy, Wolbachia trial evidence, national vector-control practice [2,4,7,14]	Municipal capacity, field teams, entomological surveillance, community acceptance, sustainability, intersectoral coordination, and financing	Hotspot coverage; cluster-response time; vector indices; household or community coverage; intervention continuity
Contextual vaccination assessment	Age-specific burden, transmission intensity, seroprevalence, circulating serotypes or lineages, vaccine safety, logistics, affordability, and pharmacovigilance capacity	Determine the need for health technology assessment, budget impact analysis, pilot evaluation, phased implementation, or non-adoption	Vaccine trial evidence, modeling studies, health technology assessment principles, and national policy alignment [15-17]	Pharmacovigilance, financing, equity, public acceptability, logistics, cold-chain capacity, national policy alignment, and uncertainty by serotype or serostatus	Coverage, adverse events, equity of access; data completeness; hospitalization trends; budget impact; cost-effectiveness in future studies
Healthcare capacity and economic risk mapping	Emergency department visits, febrile-care demand, observation capacity, beds, laboratories, fluids, workforce, referral routes, stock availability, cost domains	Hydration planning, observation capacity, laboratory prioritization, referral coordination, temporary staff redistribution, and cost-domain identification	Health system preparedness principles, Colombia-specific economic burden evidence, implementation-oriented planning [2-7]	Workforce availability, financing, geographic access, network fragmentation, service saturation, supply constraints, and data capture	Waiting time; laboratory turnaround time; observation occupancy; bed occupancy; stock availability; referral delays; resource utilization; cost domains

Conceptual architecture

**Figure 1:**
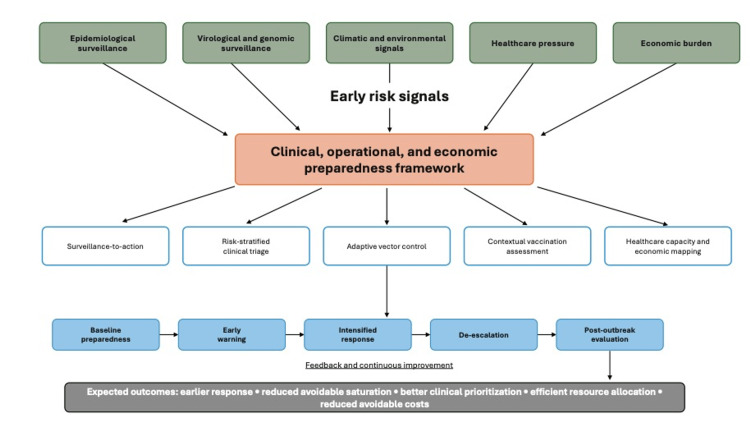
Conceptual architecture for dengue preparedness planning in Colombia Evidence-informed conceptual architecture integrating epidemiological surveillance, virological and genomic information, climatic and environmental signals, clinical severity, healthcare pressure, and economic risk mapping into five preparedness domains: surveillance-to-action, risk-stratified triage, adaptive vector control, contextual vaccination assessment, and healthcare response capacity. The figure represents a planning structure rather than a validated predictive model, implementation pathway, formal systems analysis, or economic model. The figure was created using Microsoft PowerPoint (Microsoft Corporation, Redmond, Washington, United States).

Dengue preparedness can be conceptualized as a cycle connecting risk signals, planning domains, implementation constraints, governance, and post-outbreak learning. Epidemiological surveillance, virological and genomic information, climatic and environmental signals, clinical severity, healthcare pressure, and economic burden constitute the main input streams. These inputs do not automatically produce effective action; they require defined thresholds, institutional responsibility, governance arrangements, feasible implementation pathways, and measurable indicators. The conceptual architecture is presented in Figure 1.

It was derived by grouping the main evidence streams according to their operational function. Surveillance, virological, climatic, clinical, health system, and economic inputs were positioned as upstream signals because they inform risk recognition and prioritization. The five preparedness domains were positioned as intermediate decision areas because they represent the practical points at which risk information must be translated into action. Feasibility constraints and governance requirements were positioned as modifying conditions because they determine whether planning recommendations can be implemented in real territories. Post-outbreak evaluation was incorporated as a feedback function because preparedness should be recalibrated after each epidemic peak according to observed delays, clinical outcomes, resource constraints, costs, equity gaps, and implementation barriers.

Surveillance-to-action

Surveillance should support territorial prioritization rather than function only as descriptive case reporting. In Colombia, dengue risk varies by geography, climate, vector ecology, urbanization, population mobility, local immunity, healthcare access, and institutional response capacity. Therefore, reported cases should be interpreted together with diagnostic positivity, warning-sign consultations, hospitalizations, laboratory demand, service pressure, and available virological information [6,8,9].

A pragmatic alert structure may combine four layers: routine epidemiological indicators, healthcare pressure indicators, serotype or lineage information where available, and climatic or environmental signals [3,8-11]. These layers should be interpreted as complementary inputs, not as stand-alone predictors. In high-capacity territories, integrated dashboards may combine epidemiological, clinical, laboratory, environmental, entomological, and operational data. In lower-capacity settings, simplified dashboards based on incidence, positivity, warning signs, hospitalization, referral capacity, laboratory demand, and supply availability may be more realistic. Genomic surveillance should be positioned as a complementary sentinel or reference-laboratory function rather than a universal operational requirement, because its utility depends on sample referral pathways, sequencing infrastructure, bioinformatics capacity, cost, turnaround time, and timely interpretation.

Risk-stratified clinical triage

Risk-stratified triage is the principal clinical interface between population-level preparedness and patient safety. Classification into dengue without warning signs, dengue with warning signs, and severe dengue remains central, but its operational value depends on timely identification of patients at risk of deterioration during the critical phase. Relevant clinical and laboratory features include comorbidity, persistent vomiting, abdominal pain, fluid accumulation, mucosal or gastrointestinal bleeding, lethargy, hepatomegaly, hemoconcentration with thrombocytopenia, elevated transaminases, pleural effusion, ascites, gallbladder wall thickening, and secondary infection [12].

The proposed triage structure includes three operational levels. The outpatient level applies to stable patients without warning signs, with adequate hydration, reliable follow-up, and clear return precautions. The observation level applies to patients with warning signs, comorbidities, laboratory abnormalities, social vulnerability, poor access to reassessment, or uncertain clinical trajectory. The hospital or referral level applies to suspected severe dengue, hemodynamic compromise, clinically relevant plasma leakage, significant bleeding, organ involvement, severe dehydration, or inability to ensure safe monitoring.

Biomarkers may complement clinical judgment, but should not be treated as routine operational requirements without local validation. Although C-reactive protein, aspartate aminotransferase, interleukin-8, albumin, vascular cell adhesion molecule-1, and syndecan-1 have been associated with severe manifestations [13], most low- and intermediate-complexity settings should prioritize variables that are clinically actionable, affordable, and rapidly available: warning signs, vital signs, hydration status, hematocrit, platelet count, transaminases, day of illness, comorbidities, and follow-up feasibility.

Adaptive vector control

Vector control should be planned according to micro-territorial risk rather than applied uniformly after epidemic amplification. When data allow, the operational unit should be the neighborhood, school, workplace, cluster, or high-risk block. Interventions may include breeding-site reduction, larval control, selective adulticiding, community education, active search for febrile cases, and environmental management of water storage and waste.

Feasibility depends on municipal field capacity, entomological surveillance, community participation, intersectoral coordination, and continuity beyond epidemic peaks. Adulticiding may be useful in selected circumstances, but should not replace sustained breeding-site reduction and community-based environmental management. In territories with limited operational capacity, prioritization should focus on high-transmission clusters, vulnerable populations, and areas with limited healthcare access.

Wolbachia-based strategies should be considered complementary interventions requiring contextual assessment. Although a cluster-randomized trial showed reductions in virologically confirmed dengue and dengue-related hospitalization [14], implementation in Colombia would require evaluation of ecological suitability, entomological infrastructure, community engagement, regulation, sustainability, cost, monitoring capacity, and compatibility with existing vector-control programs.

Contextual vaccination assessment

Dengue vaccination should be approached as a programmatic decision, not as a universal recommendation within this framework. TAK-003 has shown long-term efficacy and safety against virologically confirmed dengue and hospitalization, with differences according to serotype, baseline serostatus, age, and epidemiological context [15]. Modeling studies suggest that population impact may be greater in moderate- to high-transmission settings and when appropriate age cohorts are selected [16,17].

For Colombia, vaccination assessment should consider age-specific burden, transmission intensity, seroprevalence, circulating serotypes and lineages, healthcare utilization, pharmacovigilance capacity, equity, public acceptability, logistics, affordability, and competing public health priorities. Before scale-up, a formal health technology assessment, budget impact analysis, cost-effectiveness evaluation, or pilot implementation may be required. This framework does not estimate vaccine impact, economic value, or resource allocation; it identifies the decision domains that should be addressed before programmatic implementation.

Healthcare response capacity and economic risk mapping

Healthcare response capacity is central because dengue burden is expressed not only as reported cases, but also as fever consultations, laboratory demand, observation needs, hydration requirements, referral pressure, hospitalization, and follow-up. Feasible preparedness measures may include temporary febrile-care circuits, hydration areas, extended service hours, prioritized laboratory workflows, standardized triage forms, predefined referral criteria, telephone follow-up for selected patients, and temporary staff redistribution. These measures require prior governance agreements, workforce planning, financing, training, and audit indicators.

The economic component of the framework is limited to risk mapping. Its purpose is to identify cost domains relevant for future economic evaluation, including direct medical costs, direct non-medical costs, indirect costs, and institutional response costs [5]. These domains may include consultations, emergency care, laboratory testing, observation, hospitalization, fluids, medications, transportation, caregiver expenses, absenteeism, productivity loss, temporary disability, premature mortality, surveillance, vector control, risk communication, additional staff time, and temporary service reorganization.

Economic risk mapping may help identify where cost pressure is likely to emerge, but it should not be interpreted as evidence that preparedness interventions reduce costs. Future studies should quantify these assumptions through formal costing, budget impact, cost-effectiveness, or resource-allocation methods.

Feasibility and implementation constraints

Implementation feasibility is a central limitation of this framework. Integrated dashboards require interoperable data systems, standardized definitions, timely reporting, analytic personnel, and governance mechanisms. Genomic surveillance depends on sample transport, sequencing capacity, bioinformatics expertise, cost, turnaround time, and integration with epidemiological interpretation. Biomarker integration is limited by availability, affordability, standardization, and uncertainty regarding incremental clinical utility in routine care.

Workforce capacity is also critical. Triage, observation, hydration, laboratory follow-up, vector-control operations, risk communication, and data analysis require trained personnel, simplified protocols, task distribution, referral agreements, and protection of essential services. Territorial inequities further condition implementation. Rural, peri-urban, coastal, Amazonian, border, and socially vulnerable territories may differ in vector exposure, water-storage practices, healthcare access, laboratory availability, transportation times, and referral delays. Therefore, the framework should not be implemented as a single national template but adapted to local transmission patterns, institutional capacity, geography, vulnerability, and financing.

Operational activation cycle

Implementation may follow a phased cycle from baseline preparedness to early warning, intensified response, de-escalation, and post-outbreak evaluation. These phases are planning categories, not validated thresholds. Each territory should define activation criteria according to historical incidence, reporting quality, healthcare capacity, laboratory availability, governance, and available response resources. A simplified activation cycle is presented in Table 2.

**Table 2:** Proposed activation cycle for dengue preparedness planning The activation phases are conceptual planning categories and do not represent validated national thresholds. Each territory should define activation and de-escalation criteria according to historical incidence, reporting quality, laboratory capacity, healthcare service pressure, governance arrangements, and available response resources.

Phase	Activation signal	Priority action	Suggested indicator
Baseline preparedness	Inter-epidemic or expected seasonal transmission	Update triage pathways, referral routes, inventories, training, communication plans, and baseline indicators	Protocols updated; staff trained; critical inventory available; baseline indicators defined
Early warning	Increase in cases, positivity, clusters, warning-sign visits, climatic signal, laboratory demand, or vector indices	Intensify surveillance, risk communication, vector control, laboratory readiness, and outpatient triage	Signal-to-alert time; diagnostic turnaround time; cluster-response time; early warning documentation
Intensified response	Increase in consultations, observation needs, referrals, hospitalizations, severe cases, or service saturation	Expand feasible hydration and observation capacity, prioritize laboratory workflows, coordinate referrals, monitor occupancy, and redistribute staff when feasible	Waiting time; observation occupancy; referral timeliness; stock availability; laboratory turnaround time
De-escalation	Sustained reduction in incidence and healthcare pressure	Normalize services, replenish supplies, maintain focused surveillance, and preserve high-risk follow-up	Demand reduction; inventory replenishment; service continuity; residual cluster monitoring
Post-outbreak evaluation	End of epidemic peak or return to expected transmission	Audit triage, laboratory response, referrals, deaths, costs, equity gaps, and implementation barriers	Post-outbreak report; improvement plan; revised thresholds; updated preparedness actions

Explicit governance is required for each phase. Territorial health authorities should lead surveillance, vector control, community communication, intersectoral coordination, and public health alerts. Healthcare institutions should lead triage, clinical management, observation, laboratory use, patient education, referral, and patient safety. Insurers should support network coordination, authorization pathways, follow-up of at-risk patients, and analysis of service use. National authorities should provide technical guidance, surveillance standards, technology assessment processes, vaccination policy when applicable, and resource-prioritization criteria.

Future evaluation and implementation metrics

Because the proposed framework is conceptual, its operational value should be assessed through territorial piloting, stakeholder validation, and prospective implementation research. Future evaluations should include implementation, clinical, health system, equity, and economic outcomes. Implementation outcomes may include adoption, fidelity, feasibility, acceptability, reporting completeness, and signal-to-alert time. Clinical and epidemiological outcomes may include warning-sign documentation, return visits, severe dengue incidence, hospitalization rates, referral delays, and case fatality. Health system outcomes may include emergency department waiting time, observation occupancy, laboratory turnaround time, bed occupancy, stock availability, referral timeliness, and continuity of essential services. Equity outcomes should assess whether preparedness actions reach rural, peri-urban, border, coastal, Amazonian, and socially vulnerable populations. Economic outcomes should be evaluated through formal costing, budget impact analysis, cost-effectiveness analysis, or resource-allocation studies.

These metrics are intended to support future evaluation of whether integrated dengue preparedness can improve timeliness, triage performance, healthcare capacity management, equity, and economic outcomes during outbreaks. The next stage should therefore include indicator refinement, territorial adaptation, feasibility assessment, implementation testing, and prospective evaluation under real-world conditions.

## Discussion

This technical report proposes an evidence-informed conceptual framework to strengthen dengue preparedness in Colombia by integrating risk detection, territorial prioritization, clinical decision-making, prevention strategies, healthcare response capacity, governance, and economic risk mapping. Its main contribution is the organization of existing evidence and preparedness principles into an operational translation structure for a heterogeneous endemic setting. Rather than proposing a parallel dengue control strategy, the framework is intended to complement national and territorial surveillance, prevention, and response activities by clarifying how epidemiological, virological, climatic, clinical, operational, and economic inputs can be converted into preparedness decisions, feasibility assessments, and measurable indicators.

The proposed framework adds value by connecting international preparedness principles with the operational realities of dengue control in Colombia. Current World Health Organization guidance on dengue and other Aedes-borne arboviruses emphasizes preparedness, readiness, coordinated response, surveillance, clinical care, vector control, risk communication, community protection, and health system readiness [2]. Early Warning and Response System (EWARS) provides a structured approach for early warning and outbreak response based on surveillance indicators and alarm signals [3]. The PAHO Integrated Management Strategy for Arboviral Disease Prevention and Control in the Americas promotes a multidimensional model that includes patient care, epidemiological surveillance, laboratory support, integrated vector management, environmental management, communication, and intersectoral coordination [4]. These frameworks are essential references for dengue preparedness. However, their implementation requires local translation in countries where transmission risk, diagnostic capacity, municipal vector-control resources, healthcare access, referral networks, and service pressure vary substantially across territories. The present framework addresses this translational gap by organizing preparedness around practical decision points: when to activate alerts, how to triage patients safely, where to prioritize vector-control actions, how to assess preventive technologies, how to prepare healthcare services, and which cost domains should be captured for future evaluation.

In Colombia, dengue preparedness should be understood in relation to an existing surveillance and response architecture. Mandatory notification, event-specific surveillance protocols, epidemiological reporting, laboratory confirmation pathways, risk analysis, vector-control actions, and territorial response mechanisms provide an institutional platform for dengue control [6,7]. The policy and implementation challenge is therefore not the absence of dengue control components, but the degree to which these components are integrated, timely, territorially prioritized, and linked to actionable thresholds. A preparedness model becomes operationally useful when surveillance signals are connected to assigned responsibilities, feasible workflows, clinical pathways, resource planning, escalation criteria, de-escalation criteria, and post-outbreak learning.

A central implication of this framework is that dengue preparedness should move beyond single-domain responses. Surveillance without clinical readiness may detect increased transmission without reducing avoidable progression. Clinical triage without territorial intelligence may overload emergency departments and miss opportunities for targeted community action. Vector control without micro-territorial prioritization may dilute resources during epidemic amplification. Vaccination or Wolbachia-based strategies without contextual assessment may overestimate transferability from trial or modeling settings to Colombian territories. Economic burden assessment without operational linkage may quantify costs but fail to identify where potentially preventable pressure emerges. The framework, therefore, conceptualizes preparedness as an integrated decision cycle rather than as a linear sequence of isolated interventions.

Virological and genomic information illustrate the importance of contextual integration. Recent Colombian evidence has shown simultaneous circulation of multiple dengue serotypes and lineages during epidemic activity, as well as the detection of emerging genotypes such as the DENV-2 cosmopolitan genotype [8,9]. These findings support complementing routine epidemiological surveillance with virological and genomic information when available. Their value lies not only in describing viral diversity but also in informing territorial risk interpretation, outbreak investigation, and prioritization of laboratory and public health resources. However, genomic surveillance should be implemented according to capacity. Its usefulness depends on sample referral systems, sequencing infrastructure, bioinformatics capacity, cost, turnaround time, interpretive expertise, and the ability to translate findings into public health action. In many territories, sentinel or reference-laboratory approaches may be more feasible than routine genomic characterization.

Climatic and environmental signals should similarly be interpreted as preparedness inputs rather than deterministic predictors. Climate suitability, rainfall, temperature, urbanization, water-storage practices, vector ecology, and large-scale climate variability may influence dengue risk, and recent studies suggest that climate-related exposure and El Niño-Southern Oscillation dynamics may affect dengue transmission patterns [10,11]. For preparedness purposes, these signals are most useful when integrated with incidence, diagnostic positivity, case clustering, entomological indicators, healthcare pressure, and local response capacity. This distinction is important because climate-informed preparedness should strengthen risk stratification and readiness planning without replacing epidemiological surveillance, entomological assessment, or territorial judgment.

From the clinical perspective, risk-stratified triage remains the most immediately actionable component of preparedness. Warning signs and routine laboratory variables can support decisions about outpatient management, observation, hospitalization, and referral. Evidence supports the relevance of comorbidity, persistent vomiting, abdominal pain, bleeding, fluid accumulation, lethargy, hepatomegaly, hemoconcentration with thrombocytopenia, elevated transaminases, and related predictors of severe dengue [12]. Biomarkers associated with inflammation, hepatic involvement, and endothelial dysfunction may provide additional prognostic information [13], but routine incorporation into dengue triage in Colombia would require validation, affordability, timely availability, standardized interpretation, and demonstration of incremental value over clinical assessment and commonly available laboratory tests. Accordingly, the framework prioritizes variables that are clinically actionable, widely interpretable, and feasible across different levels of care.

Preventive technologies require the same contextual discipline. Wolbachia-based strategies have shown reductions in virologically confirmed dengue and dengue-related hospitalization in a cluster-randomized community trial [14]. TAK-003 has demonstrated efficacy against virologically confirmed dengue and hospitalization, although performance varies by serotype, age, baseline serostatus, and epidemiological setting [15]. Modeling studies suggest potential population-level impact in selected transmission contexts and under specific age-cohort strategies [16,17]. These findings are highly relevant for preparedness planning, but local adoption should be guided by transmission intensity, age-specific burden, seroprevalence, circulating serotypes and lineages, pharmacovigilance capacity, entomological infrastructure, equity, acceptability, financing, logistics, and alignment with national policy. In this framework, vaccination and Wolbachia-based strategies are not treated as isolated solutions, but as preventive options requiring programmatic assessment within a broader preparedness architecture.

The economic component of the framework is intentionally framed as risk mapping. This approach is appropriate because preparedness planning requires early identification of cost domains even before a formal economic evaluation is available. Relevant domains include outpatient care, emergency visits, diagnostic testing, observation, hospitalization, transportation, caregiver expenses, absenteeism, productivity loss, premature mortality, vector control, surveillance, risk communication, additional staff time, and temporary service reorganization [5]. By identifying where cost pressure may arise, economic risk mapping can guide future costing, budget impact analysis, cost-effectiveness analysis, and resource-allocation studies. This distinction protects the framework from overclaiming while preserving its policy relevance: the current model identifies economic decision domains, whereas future empirical studies should quantify whether integrated preparedness improves value, efficiency, or affordability.

Implementation feasibility is the central condition for translating the framework into practice. Integrated dashboards require interoperable information systems, standardized definitions, timely reporting, analytic personnel, and governance mechanisms. Dynamic capacity expansion requires workforce availability, financing, referral coordination, laboratory prioritization, supply management, and protection of essential services. Territorial inequities are decisive: rural, peri-urban, coastal, Amazonian, border, and socially vulnerable areas may differ in vector exposure, diagnostic access, transport times, health service availability, and referral timeliness. Therefore, the framework should not be implemented as a uniform national template. Its operational value will depend on territorial adaptation, capacity stratification, simplified tools for lower-resource settings, and governance agreements that define who acts, when, with which resources, and according to which indicators.

The framework also establishes an agenda for future evaluation. Because it was generated through structured narrative synthesis and conceptual integration, its next stage should include stakeholder validation, territorial piloting, feasibility assessment, indicator refinement, and prospective implementation research. Future studies should assess implementation outcomes such as adoption, fidelity, feasibility, acceptability, reporting completeness, and signal-to-alert time; clinical and epidemiological outcomes such as warning-sign documentation, return visits, severe dengue incidence, hospitalization rates, referral delays, and case fatality; health system outcomes such as emergency department waiting time, observation occupancy, laboratory turnaround time, bed occupancy, stock availability, and continuity of essential services; equity outcomes across vulnerable territories and populations; and economic outcomes through formal costing, budget impact analysis, cost-effectiveness analysis, or resource-allocation studies.

In summary, this framework advances dengue preparedness planning by integrating international preparedness principles, Colombia-specific operational constraints, clinical risk stratification, prevention technology assessment, health system readiness, governance, and economic risk mapping into a single conceptual structure. Its practical relevance lies in helping decision-makers move from fragmented risk signals toward coordinated, measurable, and territorially adapted preparedness actions. With stakeholder validation, territorial piloting, indicator refinement, and prospective evaluation, the framework may support more timely, equitable, and evidence-informed dengue preparedness in Colombia and may offer a structure adaptable to other heterogeneous endemic settings.

## Conclusions

Dengue preparedness in Colombia requires more than the parallel operation of surveillance, clinical care, vector control, preventive technologies, and healthcare capacity planning. In a heterogeneous endemic setting, preparedness depends on the ability to translate risk signals into timely, territorially prioritized, clinically actionable, and operationally feasible decisions. This technical report proposes an evidence-informed clinical and operational framework that integrates surveillance-to-action, risk-stratified triage, adaptive vector control, contextual vaccination assessment, healthcare response capacity, governance, and economic risk mapping within a structured preparedness cycle.

The added value of the framework lies in its function as an operational bridge between international dengue preparedness principles and the practical decision points faced by Colombian territories. By linking epidemiological, virological, climatic, clinical, health system, and economic inputs, the framework helps identify where preparedness actions should be activated, which patients and territories require prioritization, how prevention strategies should be contextually assessed, and which indicators should be monitored before, during, and after epidemic amplification.

This framework should support more coherent planning, territorial adaptation, and strategic decision-making for dengue preparedness in Colombia. Its future development should include stakeholder validation, territorial piloting, feasibility assessment, indicator refinement, implementation research, and prospective evaluation. These steps will be necessary to determine whether integrated preparedness can improve response timeliness, triage performance, healthcare capacity management, equity, and economic outcomes during dengue outbreaks.
